# Use of a highly sensitive lung cancer compact panel to detect *KRAS* G12D in the wash fluid from a lung tumor: A case report

**DOI:** 10.1111/1759-7714.14439

**Published:** 2022-05-12

**Authors:** Daisuke Minami, Nagio Takigawa, Yasuhiro Nakajima, Nobuaki Miyahara, Yasuyuki Mizumori, Mitsuhiro Ueda, Seiji Nakamura, Fumihiko Suzuki, Yoshiharu Sato, Kei Morikawa, Arihiko Kanehiro

**Affiliations:** ^1^ Department of Respiratory Medicine Himeji Saint Mary's Hospital Himeji Japan; ^2^ Department of Respiratory Medicine Hosoya Hospital Ibara Japan; ^3^ Department of Internal Medicine Himeji Saint Mary's Hospital Himeji Japan; ^4^ Department of Internal Medicine 4 Kawasaki Medical School Okayama Japan; ^5^ Department of Respiratory Medicine National Hospital Organization Himeji Medical Center Himeji Japan; ^6^ Department of Thoracic Surgery National Hospital Organization Himeji Medical Center Japan; ^7^ DNA Chip Research Inc Japan; ^8^ Division of Respiratory Medicine, Department of Internal Medicine St. Marianna University School of Medicine Kawasaki Japan

**Keywords:** *KRAS* G12D, lung cancer compact panel, pulmonary invasive mucinous adenocarcinoma

## Abstract

Here, we report a case of a pulmonary invasive mucinous adenocarcinoma harboring *KRAS* G12D, diagnosed from tumor samples containing a very small amount of tumor cells using next‐generation sequencing (NGS) and the recently developed Lung Cancer Compact Panel. A 79‐year‐old woman without any respiratory symptoms underwent chest computed tomography, which revealed a tumor in the left lower lobe. During endobronchial ultrasound (EBUS)‐guided transbronchial biopsy (TBB) using a guide sheath (GS), a sufficient specimen for pathological diagnosis could not be obtained because the patient had a severe cough and pulmonary bullae located adjacent to the tumor. In the absence of EBUS transbronchial biopsy findings using a guide sheath, brush cytology was used to categorize the tumor as class II (Papanicolaou classification). However, the wash fluid from the cytological examination contained enough cells to obtain sufficient nucleic acid for use in sequencing analysis. The latter revealed *KRAS* G12D expression. Although the patient underwent surgery without pathological evidence, the evaluation of the surgical specimen confirmed a diagnosis of pulmonary invasive mucinous adenocarcinoma. Use of the Lung Cancer Compact Panel enabled the detection of *KRAS* G12D in the wash fluid of a brush cytology sample and thus a diagnosis of pulmonary invasive mucinous adenocarcinoma.

## Graphical abstract

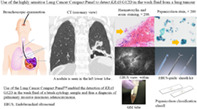

## INTRODUCTION

The detection of multiple cancer‐related genes using next‐generation sequencing (NGS), such as Foundation One CDx (Foundation Medicine, Inc.) and Oncomine Dx Target Test (Thermo Fisher Scientific), has enabled molecular targeted therapy in patients with tumors expressing *EGFR*, *ALK*, *ROS1*, *BRAF*, *MET* or *RET*.^1^ However, a frequent problem encountered is the amount of tumor cells within the specimen, as a high tumor cell content (>20%) is required to perform the panel tests. The recently approved Lung Cancer Compact Panel offers much greater sensitivity than those of other conventional NGS panels.^2^ Here, we present a case of pulmonary invasive mucinous adenocarcinoma diagnosed based on *KRAS* G12D detection using this novel panel.

### CASE REPORT

**FIGURE 1:**
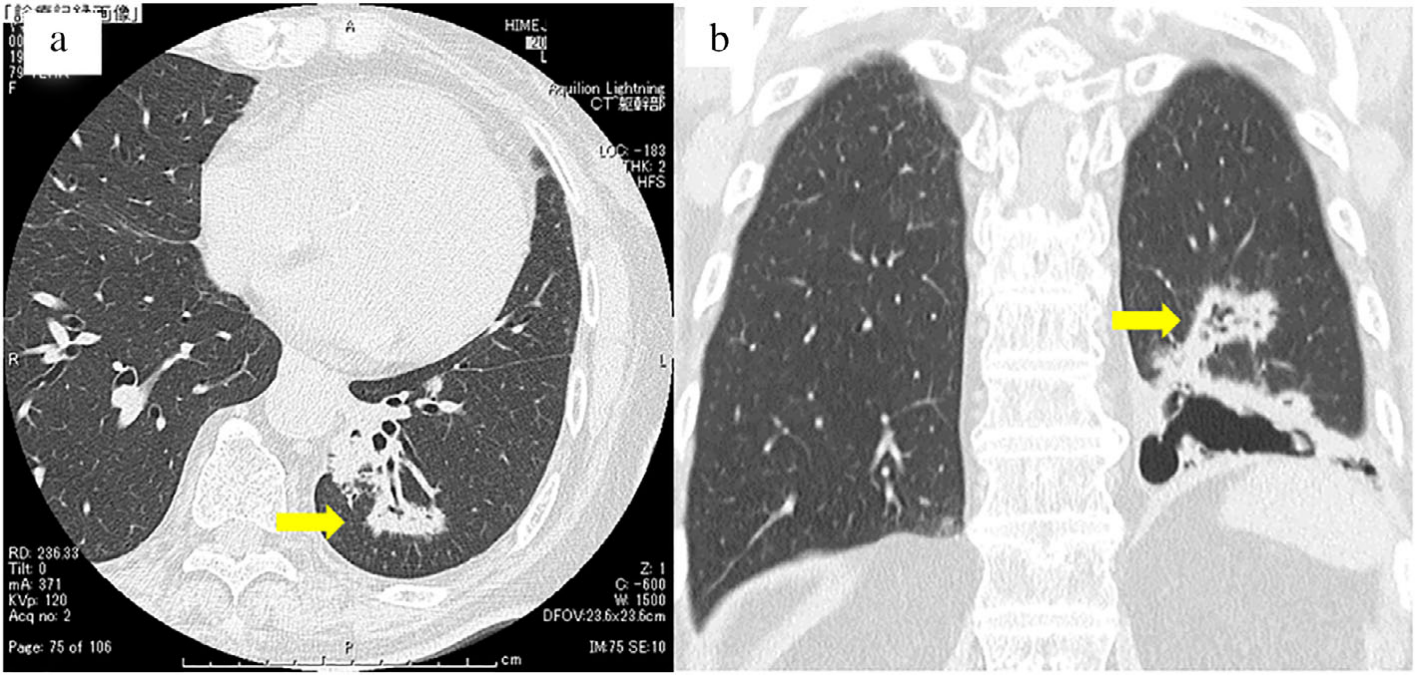
Chest computed tomography (CT) findings. (a) A nodule is seen in the left lower lobe (axial view). (b) A nodule measuring 35 mm in diameter and pulmonary bullae adjacent to the tumor can be seen (coronary view)

A 79‐year‐old woman with a 3‐year history of lumbago, diagnosed as lumbar spinal canal stenosis, was referred to our hospital for orthopedic surgery. Her chest X‐ray findings included an abnormal shadow in the left lower lung field. She had a history of hypertension, but general physical examination did not reveal any significant abnormalities. Her peripheral arterial blood oxygen saturation was 97% under room air conditions. She was a nonsmoker. Her laboratory findings were nearly normal (Table 1). Chest computed tomography (CT) revealed a mass in the left lower lobe (Figure FIGURE 1a) and pulmonary bullae adjacent to the tumor (Figure FIGURE 1b). The CT bronchus sign was detected in the tumor. Diagnostic endobronchial ultrasound (EBUS)‐guided transbronchial biopsy (TBB) using a guide sheath (GS) was performed via an endoscopic ultrasound system (EU‐ME1; Olympus) equipped with 20‐MHz mechanical radial‐type probes measuring 1.4 mm (UM‐S20‐17S; Olympus) in diameter. A thin bronchoscope (channel diameter: 2.0 mm; BF P290; Olympus) and guide sheath (external diameter: 1.95 mm; K‐201; Olympus) were used with a 1.4‐mm probe.

**TABLE 1:** Laboratory findings

Hematology	
RBC	399 × 10^4^/μl
Hematocrit	38.5%
Hb	13.2 g/dl
WBC	5.5 × 10^3^/μl
Nt.	66.4%
Lym.	28.5%
Eos.	0.7%
Bas	0.4%
Mon.	4.0%
PLT	17.5 × 10^4^/μl
Biochemistry	
Na	142 mmol/l
K	3.7 mmol/l
BUN	12 mg/dl
Cr	0.55 mg/dl
T‐Bil	0.62 mg/dl
AST	20 IU/l
ALT	17 IU/l
LDH	164 IU/l
Alb	4.0 IU/l
CRP	0.02 mg/dl
CEA	1.7 ng/ml
CYFRA	1.6 ng/ml
Pro‐GRP	65.2 pg/ml

**FIGURE 2:**
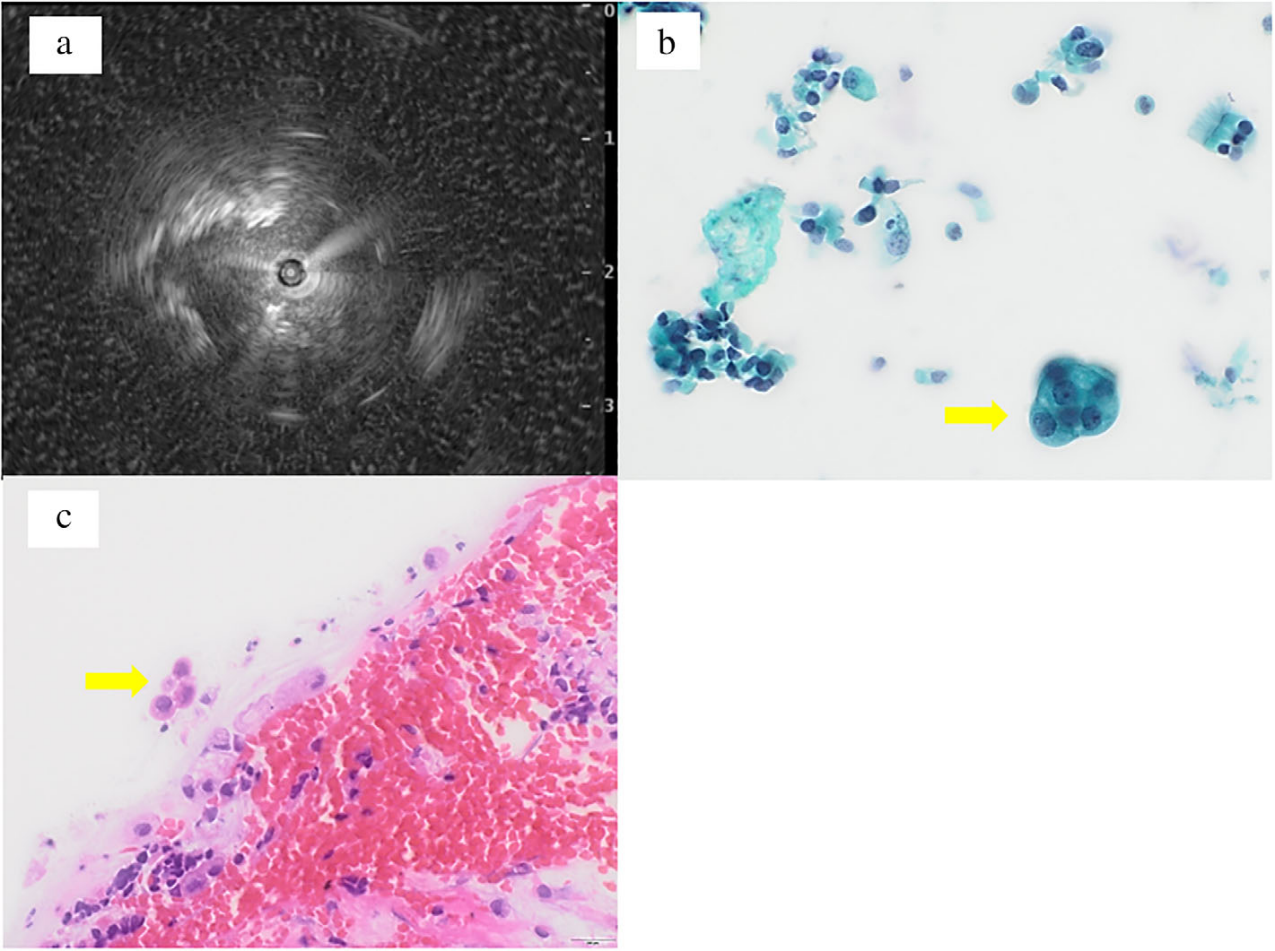
Endobronchial ultrasound‐guided transbronchial biopsy (EBUS‐TBB) using a guide sheath (GS), echo, cytological and pathological findings. (a) The echo finding shows the probe within the lesion. (b) The brush cytology specimen obtained using EBUS‐GS shows a few atypical cells (Papanicolaou staining, ×200). (c) The specimen obtained using EBUS‐GS shows a few atypical cells. Hematoxylin and eosin staining, ×200

Lidocaine (5 ml of 2% (w/v) was sprayed into the pharynx, and another 5 ml was administered through the channel during the procedures. The bronchoscope was inserted orally during conscious sedation of the patient, achieved using fentanyl (80 μg) and midazolam (6 mg). However, despite two biopsies, three brushings, and one wash during EBUS, an amount of specimen sufficient for a pathological diagnosis could not be obtained. This was most probably due to the patient's severe cough and presence of pulmonary bullae adjacent to the tumor (Figure FIGURE 1b). Histopathological examination using hematoxylin–eosin staining revealed few malignant cells. Although the brush cytology specimen was categorized as class II (Papanicolaou classification), the wash fluid from the brushing was assessed using the Lung Cancer Compact Panel because of a sufficient amount of nucleic acid (DNA 16.48 ng and RNA 196.96 ng) for this test and an EBUS view (Figure FIGURE 2a). The results revealed the presence of *KRAS* G12D, with an allele frequency of 1.3%. Moreover, all negative findings (e.g., *EGFR*, *ALK*, *ROS1*, *BRAF*, *MET*, *HER2*, and, *RET*) were examined. Based on this finding, the cells in the hematoxylin–eosin‐ and Papanicolaou‐stained samples (Figure FIGURE 2b,c) were suspected to be malignant. Therefore, the patient underwent surgery, despite a lack of pathological evidence, and the surgically resected tumor was diagnosed as invasive mucinous adenocarcinoma. The pathological stage was T4N0M0, and *KRAS* G12D was also detected in the resected tumor.

## DISCUSSION

In the US, non‐small cell lung cancer (NSCLC) samples for genetic testing are usually obtained by core needle biopsy or transthoracic fine needle aspiration biopsy, guided by CT or ultrasound.^3^ By contrast, in Japan, biopsy is mainly performed using a bronchoscope. Small peripheral pulmonary lesions can often be diagnosed using EBUS‐GS,^4^, ^5^ which is currently the most effective bronchoscopic method to collect samples from peripheral lung lesions.^6^ Nonetheless, the tumor cell content of EBUS‐GS biopsy samples may not be high enough to allow NGS using conventional panel tests. The new Lung Cancer Compact Panel permits sample analysis, including the detection of fusion genes, even when the percentage of tumor cells is very low (1%).^2^ In the present case, the specimens were classified as class II based on the observation of only a few atypical cells that could not be definitively classified as malignant. The panel analysis, however, revealed the presence of *KRAS* G12D in the tumor because of a sufficient amount of nucleic acid (DNA: 16.48 ng; RNA: 196.96 ng) from washing fluid specimen.

Pulmonary invasive mucinous adenocarcinoma is a unique variant of lung adenocarcinoma, but it is usually difficult to distinguish from infectious lung disease by imaging or bronchoscopy. Thus, in some cases, it is diagnosed in surgically resected samples. *KRAS* mutations have been linked to the clinicopathological, immunohistochemical and molecular characteristics of pulmonary invasive mucinous adenocarcinoma.^7^ Because metastatic NSCLC harboring *KRAS* G12C can be treated with KRAS‐targeting agents (e.g., sotorasib), the detection of *KRAS* subtypes using methods such as the Lung Cancer Compact Panel is essential for therapeutic decision‐making. We strongly suspected lung cancer because of this finding, and recommended surgical resection.

In our patient, transbronchial or CT‐guided transthoracic biopsy posed a risk of severe pneumothorax due to the presence of pulmonary bullae adjacent to the tumor, whereas brushing was considered to be relatively safe. To the best of our knowledge, this is the first report of the use of the Lung Cancer Compact Panel to detect *KRAS* G12D in the wash fluid from brush cytology. A prospective study on the suitability of NGS in this setting is still needed.

In conclusion, the Lung Cancer Compact Panel was effective in detecting *KRAS* G12D, and its use in analyzing gene mutations is recommended even when the tumor cell content of the specimen is low. The detection of *KRAS* mutations supports a diagnosis of pulmonary invasive mucinous adenocarcinoma.

## CONFLICT OF INTEREST

Dr Minami has received lecture fees from Hisamitsu, Taiho, AstraZeneca, and Boehringer‐Ingelheim Pharmaceuticals outside this work. Dr Takigawa has received lecture fees from Chugai Pharmaceutical and Boehringer‐Ingelheim outside this work. Dr Nakajima has received lecture fees from Ono, Chugai, Taiho, AstraZeneca, GlaxoSmithKline, Novartis, and Sanofi Pharmaceuticals. Dr Kanehiro has received lecture fees from AstraZeneca, GlaxoSmithKline, Novartis, Boehringer‐Ingelheim and Sanofi Pharmaceuticals outside this work. Dr Miyahara has received lecture fees from AstraZeneca, GlaxoSmithKline, Novartis, Boehringer‐Ingelheim, Kyorin and Sanofi Pharmaceuticals outside this work. Dr Mizumori has received lecture fees from Chugai, AstraZeneca, Novartis, Sanofi Pharmaceuticals, and AMC corporation outside this work. Dr Morikawa has received lecture fees from AstraZeneca, Boehringer‐Ingelheim Pharmaceuticals and Chugai Pharmaceutical Co., Ltd outside this study. The remaining authors declare no competing interests.
